# On the Internal Administration of Chloroform in Delirium Tremens

**Published:** 1852-12

**Authors:** Richard G. H. Butcher

**Affiliations:** Surgeon to Mercer’s Hospital, &c.


*On the Internal Administration of Chloroform in Delirium Tremens.* By Richard G. H. Butcher, F. R. C. S. I., Surgeon to Mercer's Hospital, &c.—The patient, Wm. Magrath, aged 26, a powerful young man, by trade, a wine porter, was admitted by the author, into Mercer's Hospital, June 25th, 1852. When admitted, four days had elapsed from the time of his giving up the stimulus, and he had no sleep during that period. His countenance was particularly anxious, with a wild expression: the pathognomonic symptom, tremor of the tongue and hands fully established. His speech was hurried and uneven; he was quite irrational and wild; pulse 120; surface of the body hot and burning, while his face was covered with perspiration, and his hair drenched in sweat. He was put into bed but would nat remain quiet; got up and kept constantly walking up and down the ward and corridor. He was ordered two grains of calomel and a grain of opium in pill, to be taken every third hour. He had taken three, but each was vomited almost immediately after being swallowed. A draught containing one grain of morphine, two drops of creosote, and an ounce of camphor mixture, to be given every third hour, was next tried, but this was likewise rejected. If the patient only took a sup of cold water to moisten his parched mouth and lips, it was instantly vomited.

On the following morning, the 20th, his condition was a great deal worse, and the case now assumed a serious aspect. From the irritability of the stomach, opium in any form could not be got to rest upon it. As for the idea of administering repeated small opiate enemata in this powerful, restless, and uncontrollable young man, the practicability of it could not be entertained for a moment. From the satisfactory issue of two cases, reported in the *American Journal of Medical Science* for January, 1852, the same practice was determined on—the internal administration of chloroform.

At ten o'clock this morning, (26th,) one drachm of pure chloroform in two ounces and a half of water was administered. In an hour after swallowing it, the patient became comparatively tranquil, and could be persuaded to lie in bed.

Eleven o'clock:—He began to get drowsey, and slept for periods of from ten to twelve minutes at a time. At a quarter before one o'clock, he became fully affected by the medicine, and fell into a quiet steady sleep; and on visiting him at two and four p. m. he was still in a profound sleep, and continued so until seven in the evening. During this long sleep of six hours, he was calm and quiet; his pulse fell from 120, which it was in the morning, to 96, at which it remained; his respirations were between 16 and twenty in the minute, and not louder than natural; the temperature of the body was exalted. All along heat was maintained to the feet, and a pure current of air circulating around him, the windows being kept open. On his awakening, he was nearly or quite sensible, and advantage was taken of this pause to administer a full stimulant cathartic, consisting of six grains of calomel and ten grains of camphor, not only with the intention of freeing the bowels of accumulated matter, but likewise to guard against congestion of the brain. Orders were left in case he should not sleep before ten, to administer half a drachm of chloroform in two ounces of camphor mixture.

27th, ten A. M.—The patient went to sleep almost immediately after swallowing the bolus on last evening, so he did not require the chloroform draught. His bowels were opened three times very freely during the night, and his condition is in every way greatly improved. He is quite rational, and answers every question sensibly; his pulse 96, considerable volume; skin cool; after being interrogated, he quietly turned on his side and went to sleep.

Three P. M.—His bowels have been several times opened since morning, yet his pulse has risen to 110; the temperature of his body is also increased; he is hot and burning; altogether he is excited, and the fear of horrible objects around him has returned. On the presence of those symptoms the chloroform draught was at once repeated. Shortly after, he took a large drink of tea, which was inadvertantly left beside his bed, which produced vomiting immediately.

Nine P. M.—Since the last visit, the patient has slept at short intervals, for one and two hours at a time; pulse still up to 110. Ordered the chloroform draught, one drachm to two ounces and a half of the camphor mixture, to be repeated.

28th:—After the patient had taken the draught last night, he fell into a quiet sleep, which continued uninterrupted until eight o'clock this morning. He awoke quite collected and rational; his pulse 80; skin cool; his tongue and extremities quite free from tremor, and he feels in every respect well; his appetite has returned, and all food is retained on the stomach. Ordered a grain of morphia in an ounce of camphor mixture, to be given at night.

29th:—This morning the patient is quite restored; he is sitting up eating his breakfast heartily in bed; in short, he is quite convalescent, and only requires a little nourishment to remove the debility consequent upon so severe a struggle.

In reference to the administration of chloroform in the foregoing case remarks the author in conclusion, there is one point which solicits our closest attention, namely: the remarkable lowering of the pulse, when the perfect effect of the medicine was produced; the pulse, in fact, might form the index to direct the practitioner as to the propriety of a repetition of the dose. Again as a precautionary measure, I consider it desirable to keep the heat to the feet, and a current of pure air circulating around the bed and through the apartment in which the patient lies.—*Dublin Medical Press.*

